# MiR-424-5p acts as an oncogene in Hep3B cells by activating the PI3K/AKT signaling pathway

**DOI:** 10.3389/fonc.2025.1726596

**Published:** 2026-01-26

**Authors:** Zhixian Ding, Shuaiyong Qi, Mengxue Hu, Lusheng Wang, Yu Tang, Jiting Sun, Lijie Zheng, Yafen Li, Lili Li, Quangen Ma, Heng Tang

**Affiliations:** 1 Central Laboratory, Wanbei Coal Electric Group General Hospital, Suzhou Anhui, China; 2 MGI Tech Co., Ltd, Guangdong Shenzhen, China

**Keywords:** HBV, Hep3B cells, hepatocellular carcinoma, miR-424-5p, PI3K/Akt pathway

## Abstract

Hepatocellular carcinoma (HCC) remains a global health challenge with high morbidity and mortality. MicroRNAs (miRNAs) play pivotal roles in cancer progression, yet their context-dependent functions in HBV-HCC are unclear. This study demonstrates that miR-424-5p is significantly upregulated in HBV positive Hep3B cells, correlating with poor patient prognosis. Integrated bioinformatic analysis predicted that the target genes of miR-424-5p are significantly enriched in the PI3K/AKT signaling pathway. Functional experiments showed that knockdown of miR-424-5p suppressed cell proliferation, migration, and colony formation. Mechanistically, miR-424-5p knockdown led to the upregulation of PTEN and downregulation of phosphorylated PI3K/AKT, indicating inhibition of this pathway. These findings unveil an oncogenic role of miR-424-5p in HBV-HCC, suggesting its function is driven by viral specific dysregulation of the PI3K/AKT pathway, with PTEN involvement. Our study highlights miR-424-5p as a potential therapeutic target and provides insights into etiology-specific miRNA regulatory networks.

## 1 Introduction

HCC remains a major global health challenge, ranking as a leading cause of cancer-related death worldwide (1, 2). Its high mortality is attributed to frequent late-stage diagnosis, limited curative options for advanced disease, and high rates of recurrence and metastasis. A critical aspect of HCC heterogeneity is its diverse etiology, with chronic hepatitis B virus (HBV) infection being one of the most prominent risk factors globally. HBV-associated HCC (HBV-HCC) often exhibits distinct molecular pathogenesis compared to HCCs arising from other causes, such as altered signaling pathways and unique patterns of viral-host genomic interactions (3, 4). Despite significant advances in diagnosis and therapy, the underlying molecular mechanisms, particularly those specific to viral etiologies like HBV, remain incompletely elucidated. This gap underscores the necessity of identifying novel, etiology-specific therapeutic targets and molecular drivers.

miRNAs are a class of small non-coding RNA molecules that play crucial roles in post-transcriptional gene regulation by binding to complementary sequences within messenger RNAs (mRNAs) (5, 6). Aberrant miRNA expression has been implicated in various human diseases. Particularly in cancer, miRNAs can act as either oncogenes or tumor suppressor genes, influencing tumor initiation, proliferation, invasion, and metastasis (7, 8).

The role of miRNAs is particularly compelling in the context of HBV-HCC. The HBV virus, especially through its regulatory protein HBx, can significantly reshape the host cell’s transcriptome, including the expression profiles of numerous miRNAs (9). This viral-mediated dysregulation can subvert normal cellular miRNA functions, driving oncogenic processes in a manner distinct from non-viral HCC (10). However, the precise mechanisms by which HBV infection alters the functional output of specific miRNAs, potentially converting them from tumor suppressors to oncogenes, remain a critical area of investigation.

Against this backdrop, the miR-424/503 cluster, and specifically miR-424-5p, presents an intriguing case study.Notably, miR-424, a member of the miR-424/503 cluster, has been reported to be dysregulated in various cancers and exhibits context-dependent roles. For instance, it functions as an oncogene by promoting cell proliferation, migration, and invasion in breast cancer (11), colorectal cancer (12), and lung cancer (13). Conversely, it may act as a tumor suppressor in other contexts (14). In HCC, the role and regulatory mechanisms of miR-424-5p are primarily characterized as tumor-suppressive. This functional duality is particularly intriguing in HCC. In non-viral or HBV-negative contexts, miR-424-5p is well-established as a tumor suppressor. For instance, it inhibits epithelial-mesenchymal transition (EMT) by targeting ICAT (15–17)and suppresses angiogenesis by targeting VEGFR2 (18, 19).This well-established tumor-suppressive role in non-viral contexts makes the following observation particularly striking. However, an aberrant upregulation of miR-424-5p has been observed in HBV positive HCC clinical samples and cell lines (20).​ This paradox indicates that viral infection, specifically HBV, may fundamentally alter the functional output of this miRNA, potentially through unique molecular alterations (e.g., dysregulation of non-coding RNAs) resulting from viral integration (21, 22). The stark contrast between its reported tumor-suppressive role and its observed upregulation in HBV-HCC underscores a critical knowledge gap. Given the distinct pathological features of HBV-HCC, this study specifically aims to elucidate the role and mechanism of miR-424-5p using the HBV-integrated Hep3B cell line, a classic model for HBV-HCC research, to uncover its virus-specific oncogenic function.

To validate this possibility, we conducted a comprehensive study aimed at analyzing the expression of miR-424-5p in HCC cell lines and elucidating its functional consequences and underlying molecular mechanisms. In this study, we first performed whole-transcriptome sequencing of the normal hepatocyte line L02 and the HCC cell line Hep3B, which revealed a significant upregulation of miR-424-5p in Hep3B cells. KEGG/GO pathway enrichment analysis indicated the strongest correlation with the PI3K-Akt signaling pathway.

The PI3K-Akt signaling pathway (phosphatidylinositol 3-kinase/protein kinase B signaling pathway) is a critical signal transduction network within cells, extensively involved in regulating fundamental biological processes such as cell growth, proliferation, survival, metabolism, and migration. The core component of this pathway is PI3K, which phosphorylates PI (4, 5)P2 to generate PI (3–5)P3. Acting as a second messenger, PI (3–5)P3 recruits and activates downstream Akt (also known as PKB). Akt mediates a variety of cellular physiological functions by phosphorylating a series of substrate proteins. PTEN (phosphatase and tensin homolog), a negative regulator of the PI3K-Akt pathway, dephosphorylates PI (3–5)P3, thereby inhibiting PI3K-Akt pathway activity. Consequently, the inactivation of PTEN or aberrant activation of the PI3K-Akt pathway has been widely observed in various cancers and represents a key event driving tumorigenesis and progression (23, 24).To further validate the impact of miR-424-5p on the PI3K-Akt pathway and its downstream cellular functions, we performed multiple experiments, including detecting the expression levels of key pathway proteins (Akt, p-Akt, and PTEN) and conducting functional assays for cell proliferation and migration.

Our findings robustly demonstrate that miR-424-5p exerts a critical oncogenic role in HCC by activating the PI3K-Akt signaling pathway, significantly promoting cancer cell proliferation and migration. This study provides novel insights into the molecular pathogenesis of HCC and highlights the potential of miR-424-5p as a therapeutic target for HCC.

## 2 Materials and methods

### 2.1 Cell culture

The human hepatocellular carcinoma cell line Hep3B and the normal human hepatocyte line L02 were purchased from the Shanghai Institute of Cell Biology, Chinese Academy of Sciences (Shanghai, China). All cell lines were cultured in Dulbecco’s Modified Eagle Medium (DMEM; Gibco, Grand Island, NY, USA) supplemented with 10% fetal bovine serum, 100 U/mL penicillin, and 100 μg/mL streptomycin. Cells were maintained at 37°C in a humidified atmosphere containing 5% CO_2_.

### 2.2 Whole-transcriptome sequencing and data analysis

L02 and Hep3B cells were collected and suspended in Trizol reagent (Invitrogen, Thermo Fisher Scientific, USA) according to the manufacturer’s instructions. Total RNA was extracted and quantified using an ND-2000 spectrophotometer (NanoDrop Technologies,USA). RNA quality was assessed based on the following criteria: OD260/280 ratio between 1.8 and 2.2, OD260/230 ratio ≥ 2.0, RNA Integrity Number (RIN) ≥ 8.0, 28S:18S ratio ≥ 1.0, and total RNA amount ≥ 1 μg to ensure high-quality RNA for library construction. RNA purification, sequencing, and library preparation were performed by Shanghai Majorbio Bio-pharm Technology Co., Ltd. (Shanghai, China) following the manufacturer’s protocols (Illumina, San Diego, CA, USA).Sequencing data were analyzed using the DESeq2, edgeR, and Limma packages. Genes with an adjusted P-value (P-adjust) < 0.05 after multiple testing correction (e.g., Benjamini–Hochberg method) were considered significantly differentially expressed genes (DEGs). To visualize the statistical significance and magnitude of expression changes, volcano plots were generated with the x-axis representing log_2_ Fold Change and the y-axis representing -log_10_(P-adjust value).Functional annotation of DEGs was performed using the Gene Ontology (GO) database (http://www.geneontology.org), and GO term enrichment analysis was conducted with DAVID (https://davidbioinformatics.nih.gov/). KEGG (Kyoto Encyclopedia of Genes and Genomes) pathway enrichment analysis was also carried out using DAVID to identify significantly enriched pathways (P-adjust ≤ 0.05).

### 2.3 Cell transfection

Cell transfection was performed using the siRNA-mate plus transfection reagent kit (Genomeditech, Shanghai, China). Hep3B cells were seeded into 6-well plates and transfected with siRNA when cell density reached 60–70% confluence, following the manufacturer’s protocol. Cells were divided into two groups: the negative control group (NC group), transfected with a non-targeting siRNA, and the miR-424 inhibitor group (in-miR-424 group), transfected with an inhibitor designed to suppress miR-424 activity. After 48 h of transfection, cells were harvested for subsequent RNA extraction, protein detection, and functional assays.

### 2.4 Quantitative real-time PCR

Total RNA was extracted using TRIzol reagent (Invitrogen, Thermo Fisher Scientific, USA). Reverse transcription of mRNA was performed using the cDNA Synthesis Kit (TaKaRa), while miRNA was reverse transcribed using a stem-loop method-based miRNA RT Kit (Sangon Biotech, Shanghai, China), strictly following the manufacturers’ protocols.Gene expression was detected using a LightCycler 480 Real-Time PCR System (Roche). The reaction conditions followed the instructions of the SYBR Green Mix (Roche). The thermal cycling parameters were set as follows: pre-denaturation at 95°C for 10 min; 45 cycles of denaturation at 95°C for 15 s, annealing at 60°C for 20 s, and extension at 72°C for 30 s; final extension at 72°C for 5 min. Each reaction was performed in triplicate to ensure reliability. U6 and GAPDH were used as internal reference genes for miRNA and mRNA normalization, respectively.

Data analysis was conducted using the 2^(-ΔΔCt) method. The calculations were performed as follows: ΔCt = Ct (target gene) - Ct (reference gene); ΔΔCt = ΔCt (experimental group) - ΔCt (control group). The primer sequences for the target genes and their internal references are listed in **Table 1**.

**Table 1:** Primer sequences.

Name of primer	Sequences
miR-424-5p-F	CAGCAGCAATTCATGT
miR-424-5p-R	TGGTGTCGTGGAGTCG
U6-F	CTCTCGCTTCGGCAGCACA
U6-R	ACGCTTCACGAATTTGCGT
STXBP5-F	GCTGGAAGTGGTTCCGTACAT
STXBP5-R	GAACTGGATCAAAGGCTAATGCT
Fsd1L-F	AAGGAAAGCATGATTAGCACCA
Fsd1L-R	TGACTGAGCTGACTCTGTAACT
SYMPK-F	GATGGTAAAGTCACGGGTCATT
SYMPK-R	GTCCAATAGCAGGATGATGTCC
SAMD4A-F	CAGAAGCTCTTTCGGTCTTTCC
SAMD4A-R	AAGCCTCGATTTCTTTGCTGT
TLL1-F	AAATCGAGTTCCCAGAGCCG
TLL1-R	TCATCACGGGAGGGGAGAAT
TP53BP1-F	ATGGACCCTACTGGAAGTCAG
TP53BP1-R	TTTCTTTGTGCGTCTGGAGATT
GAPDH-F	AGTCCACTG GCGTCTTCA
GAPDH-R	GAGTC CTTCCACGATACCAA

### 2.5 Cell counting kit-8 assay

Cell proliferative ability was measured using the CCK-8(Beyotime Biotechnology, Shanghai, China). Cells from different treatment groups were seeded into 96-well plates at a density of 3×10³ cells per well. Then, 10 µL of CCK-8 solution was added to each well. The optical density (OD) values at 450nm were recorded using a spectrophotometer at 0, 24, 48, and 72 h. Each experiment was performed in triplicate.

### 2.6 Colony formation assay

Hep3B cells were seeded into 6-well culture plates at a density of 500 cells per well and cultured at 37°C with 5% CO_2_ for 14 days. After incubation, the medium was discarded, and the cells were washed twice with PBS. Subsequently, the cells were fixed with 4% paraformaldehyde at room temperature for 15 min and stained with 0.1% crystal violet at room temperature for 30 min. After staining, the cells were rinsed with water until dry, then photographed, and colonies (defined as cell clusters with a diameter >0.5 mm and containing at least 50 cells) were counted.

### 2.7 Transwell assay

Hep3B cells were resuspended in 100 µL of serum-free medium at a density of 5×10^4^ cells per well and added to the upper chamber of a Transwell insert (8 µm pore size). The lower chamber was filled with 600-800 µL of medium containing 10% FBS. The plates were incubated at 37°C with 5% CO_2_ for 24 h. After incubation, the Transwell insert was removed. Cells were fixed with 4% paraformaldehyde at room temperature for 15 min and then stained with 0.1% crystal violet at room temperature for 30 min. Non-migratory cells on the interior of the upper chamber were removed using a cotton swab. The migrated cells on the bottom surface of the membrane were photographed and counted.

### 2.8 Cell scratch assay

Hep3B cells were seeded into 6-well plates and cultured until they reached high confluence (approximately 90%-100%). A straight scratch was created in the cell monolayer using a pipette tip. The medium was then replaced with low-serum medium (containing 1% FBS). Cells were incubated at 37°C with 5% CO_2_ for 24–48 h. Images of the wound area were captured under a microscope at 0, 24, and 48 h. The change in wound width was measured to assess cell migration.

### 2.9 EdU cell proliferation assay

Hep3B cells were seeded into 24-well plates at a density of 5×10^4^ cells per well. After 24 h of culture, a working concentration of EdU solution (10 µM) was added to the cells, followed by a further 2 h incubation. After EdU labeling, subsequent cell processing steps were performed according to the instructions of the EdU detection kit (C10310-1, Ribobio,China), which included: cell fixation (typically using 4% paraformaldehyde), cell permeabilization (typically using 0.5% Triton X-100), and the Click reaction to label the EdU incorporated into newly synthesized DNA (typically producing green fluorescence). Finally, the cells were observed and imaged under a fluorescence microscope. The percentage of EdU-positive cells (green fluorescence) relative to the total number of DAPI-stained nuclei (blue fluorescence) was counted to calculate the EdU-positive rate (%).

### 2.10 Cell cycle analysis

Cell cycle distribution was analyzed by flow cytometry. Following transfection and treatment (including administration of the AKT activator SC79, 305834-79-1, MedChemExpress), Hep3B cells were harvested by trypsinization, washed with cold PBS, and fixed in 70% ice-cold ethanol at 4°C overnight. Fixed cells were washed with PBS and then stained in the dark with a solution containing RNase A (100 μg/mL; Beyotime Biotechnology, China) and propidium iodide (PI, 50 μg/mL; Beyotime Biotechnology, China) at 37°C for 30 min. Cell cycle profiles (G0/G1, S, and G2/M phases) were analyzed using a BD FACSCalibur flow cytometer. Data from at least 10,000 events per sample were collected and analyzed using FlowJo software (version 10.8). All experiments were performed in triplicate.

### 2.11 Western blot analysis

Cells were lysed using RIPA lysis buffer (P0013B, Beyotime) to obtain protein samples. After measuring protein concentration with a BCA kit (P0011, Beyotime), an appropriate volume of protein was mixed with loading buffer (P0015, Beyotime) and denatured by boiling in a water bath for 10 min. Electrophoresis was initially performed at 80 V for 30 min. After the bromophenol blue dye entered the separation gel, the voltage was switched to 120 V until proteins were adequately separated.

Membrane transfer was conducted in an ice bath at a constant current of 300 mA for 88 min. Following transfer, the membrane was briefly rinsed in TBST washing buffer for 1–2 min and then blocked with 5% BSA blocking buffer at room temperature for 2 h. The membrane was incubated with primary antibodies overnight at 4°C on a shaking platform. The primary antibodies used were: β-Actin (66009-1-Ig, 1:20000), AKT (60203-2-Ig, 1:5000), p-AKT (66444-1-Ig, 1:5000), PI3K (67071-1-Ig, 1:1000), and PTEN (60300-3-Ig, 1:1000) from Proteintech (USA); and p-PI3K (4249T, 1:1000) from CST (USA).

The next day, the membrane was washed three times with TBST, 10 min each time. Subsequently, it was incubated with horseradish peroxidase-conjugated goat anti-rabbit IgG secondary antibody (1:5000, ComWin Biotech, Beijing) at room temperature for 1.5 h, followed by three additional TBST washes (10 min each). After adding developing solution, protein bands were detected using a chemiluminescence imaging system (Tanon 5200).

### 2.12 Statistical analysis

Statistical analyses were performed using GraphPad Prism 7.0 software. All data are presented as mean ± standard deviation. Comparisons between two groups were conducted using Student’s t-test, while one-way analysis of variance (ANOVA) was employed for comparisons among multiple groups. Correlation analyses were performed using Pearson’s correlation coefficient. Survival analysis was carried out using the Kaplan-Meier method. A p-value of less than 0.05 (*P* < 0.05) was considered statistically significant.

## 3 Results

### 3.1 miR-424-5p is significantly upregulated in hep3b cell and associated with poor prognosis

**Figure 1:**
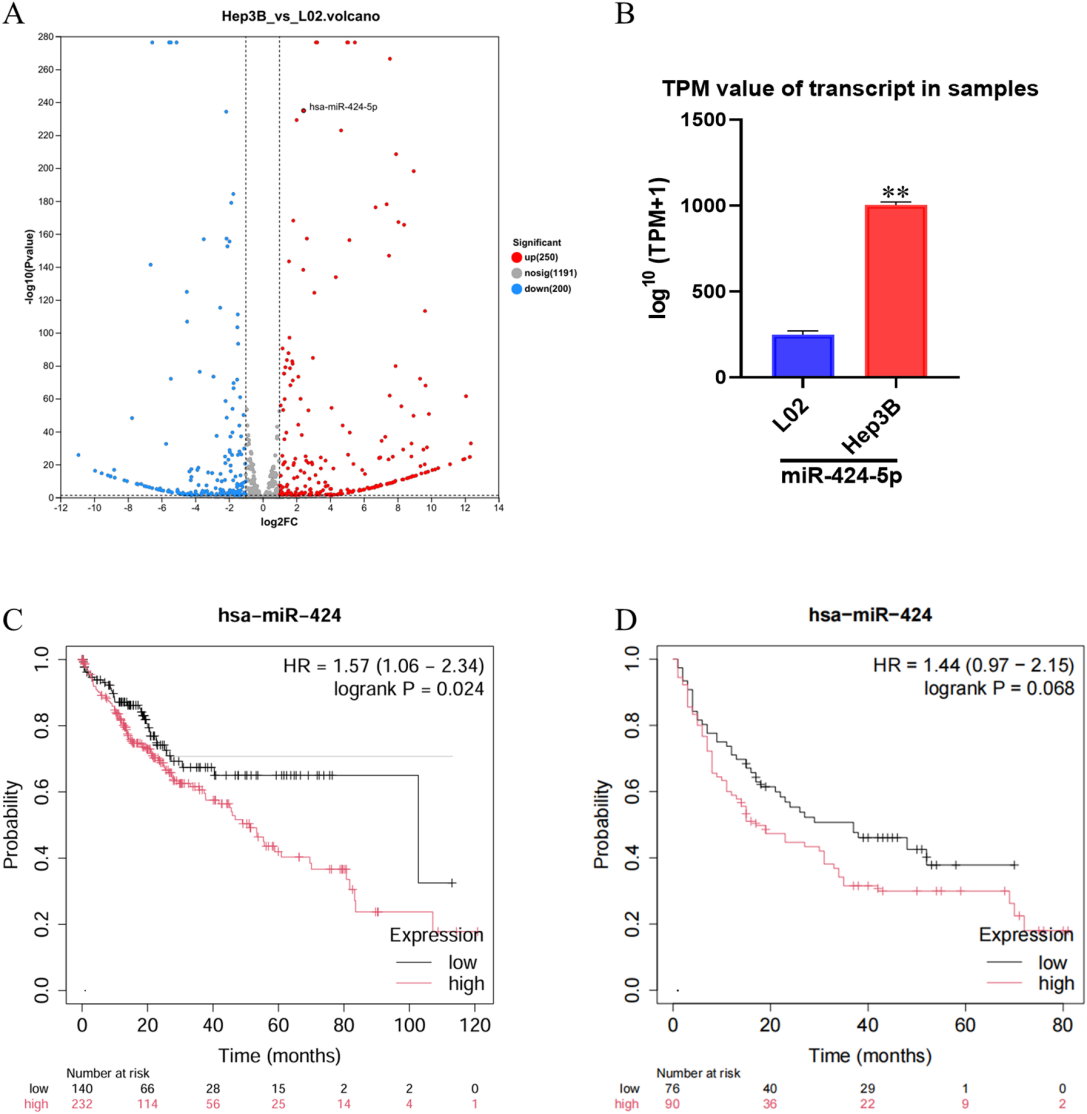
Expression of miR-424-5p in Hep3B cells and its impact on patient survival. **(A)** Volcano plot of DEGs between Hep3B and L02 cells. Red: significantly upregulated genes (log_2_^FC^ > 1, p < 0.05); blue: significantly downregulated genes (log_2_^FC^ < -1, p < 0.05); gray: non-significant genes. **(B)** Expression level of miR-424-5p in L02 and Hep3B cells, presented as log_2_^(TPM + 1)^. Data are mean ± SD. ^**^p < 0.01. **(C, D)**: Kaplan-Meier survival analysis of HCC patients stratified by miR-424 expression (high vs. low). HR and log-rank p-value are shown.

Whole-transcriptome sequencing analysis revealed significant gene expression differences between Hep3B and LO2. Volcano plot analysis identified a total of 1,248 differentially expressed genes (|log_2_FC| > 1.0, *P* < 0.05), among which miR-424-5p demonstrated the most pronounced upregulation in Hep3B cell (**Figure 1A**). Transcripts per million (TPM) quantification further confirmed that the expression level of miR-424-5p in Hep3B cell was significantly higher than that in LO2 cell (P < 0.001) (**Figure 1B**).

To evaluate the clinical significance of these findings, survival analysis was performed. The results demonstrated that patients with high miR-424 expression had significantly shorter overall survival compared to those with low expression (Hazard Ratio [HR] = 1.57, 95% Confidence Interval [CI]: 1.06–2.34; P = 0.024) (**Figure 1C**). Although a similar trend was observed in an independent validation cohort (HR = 1.44, 95% CI: 0.97–2.15), the difference did not reach statistical significance (P = 0.068) (**Figure 1D**), which may be attributable to limited sample size or inherent cohort heterogeneity.

This prominent upregulation of miR-424-5p specifically in the HBV-positive Hep3B cells, coupled with its association with poor patient survival, prompted us to focus subsequent functional and mechanistic investigations on this specific cellular context.​ We hypothesized that in the setting of HBV infection, miR-424-5p might acquire a distinct, oncogenic role.

### 3.2 Identification and validation of miR-424-5p target genes

**Figure 2:**
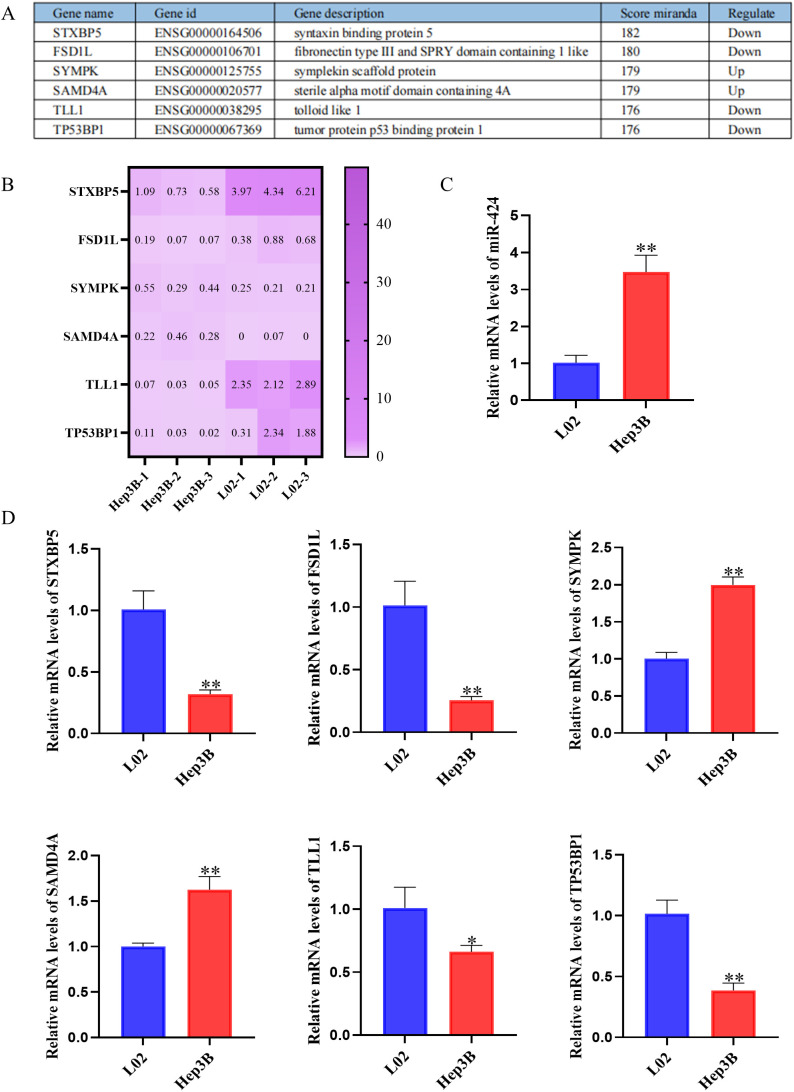
Predicted target genes of miR-424 and their expression in Hep3B cells. **(A)** Top predicted target genes of miR-424, including gene name, ID, description, Miranda score, and regulation direction. **(B)** Heatmap of selected target genes (STXBP5, FSD1L, SYMPK, SAMD4A, TLL1, TP53BP1) in Hep3B and L02 cells. **(C)** Relative mRNA level of miR-424 in L02 and Hep3B cells by qRT-PCR. Data are mean ± SD(n=3). ^**^p < 0.01. **(D)** Relative mRNA levels of predicted target genes in L02 and Hep3B cells by qRT-PCR. Data are mean ± SD(n=3). ^*^p < 0.05, ^**^p < 0.01.

Given the significant upregulation of miR-424-5p in Hep3B cell and its association with poor prognosis, we further investigated its functional role and downstream molecular mechanisms. Based on whole-transcriptome sequencing analysis, six candidate target genes with the highest binding scores (Miranda score: 176-182) were selected from the downstream targets of upregulated miR-424-5p in Hep3B cells, including STXBP5, FSD1L, SYMPK, SAMD4A, TLL1, and TP53BP1. These six genes showed significant differential expression between the Hep3B cells and the L02 cells (**Figures 2A, B**).

Subsequently, qPCR validation was performed to examine the expression levels of miR-424-5p and these target genes in Hep3B cells. The results demonstrated that the expression levels of miR-424-5p, SYMPK, and SAMD4A were significantly higher in Hep3B cells compared to L02 cells (*P* < 0.01), while STXBP5, FSD1L, TLL1, and TP53BP1 showed significantly reduced expression (*P* < 0.01) (**Figures 2C, D**). These findings are consistent with the transcriptomic predictions of target gene expression. These results not only validate the reliability of the bioinformatic predictions but also reveal that miR-424-5p may regulate the expression of downstream target genes through a complex network in HCC.

### 3.3 Knockdown of miR-424-5p inhibits the migratory ability of Hep3B cells

**Figure 3:**
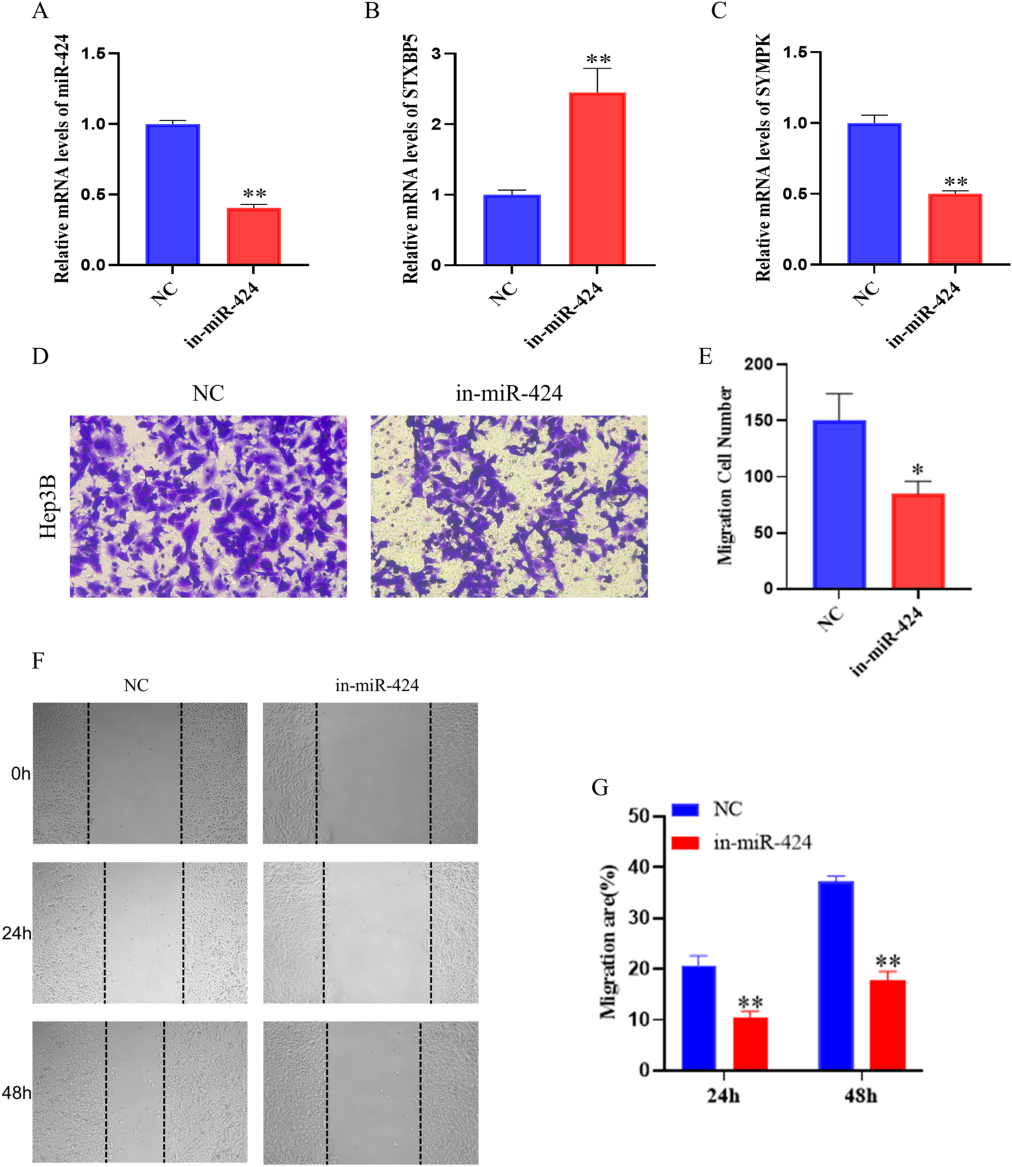
Effect of miR-424 inhibition on migration and invasion of Hep3B. **(A)** Relative mRNA level of miR-424 in Hep3B cells transfected with in-miR-424 or NC. Data are mean ± SD(n=3). ^**^p < 0.01. **(B)** Relative mRNA level of STXBP5. Data are mean ± SD(n=3). ^**^p < 0.01. **(C)** Relative mRNA level of SYMPK. Data are mean ± SD(n=3). ^**^p < 0.01. **(D)** Representative images of transwell migration assay. **(E)** Quantification of migrated cells. Data are mean ± SD(n=3). ^*^p < 0.05. **(F)** Representative images of wound healing assay at 0, 24, and 48 hours. **(G)** Quantification of migration rate. Data are mean ± SD(n=3). ^**^p < 0.01.

Based on previous findings, we further investigated the effect of miR-424-5p on the migratory ability of Hep3B Cells. We first verified the knockdown efficiency of miR-424-5p by qPCR (**Figure 3A**), which showed a significant reduction in miR-424-5p expression levels in the knockdown group compared to the control group (*P* < 0.01).

Subsequently, we examined the expression changes of two key target genes, STYXPS and SYMPK (**Figures 3B, C**). The results demonstrated that following miR-424-5p knockdown, STYXPS expression was significantly upregulated (*P* < 0.01), while SYMPK expression was significantly downregulated (*P* < 0.01). These findings are entirely consistent with the bioinformatic predictions.

To assess the impact of miR-424-5p on the migratory capacity of HCC cells, we conducted Transwell and wound healing assays. The Transwell assay results revealed a significant decrease in the number of migrating cells after miR-424-5p knockdown compared to the control group (*P* < 0.05) (**Figures 3D, E**). The wound healing assay further confirmed that the wound closure rates at both 24 and 48 h were significantly lower in the knockdown group than in the control group (*P* < 0.01) (**Figures 3F, G**).These results collectively indicate that miR-424-5p plays a crucial role in the migration process of Hep3B cells.

### 3.4 Knockdown of miR-424-5p significantly suppresses the proliferative capacity of Hep3B cells

**Figure 4:**
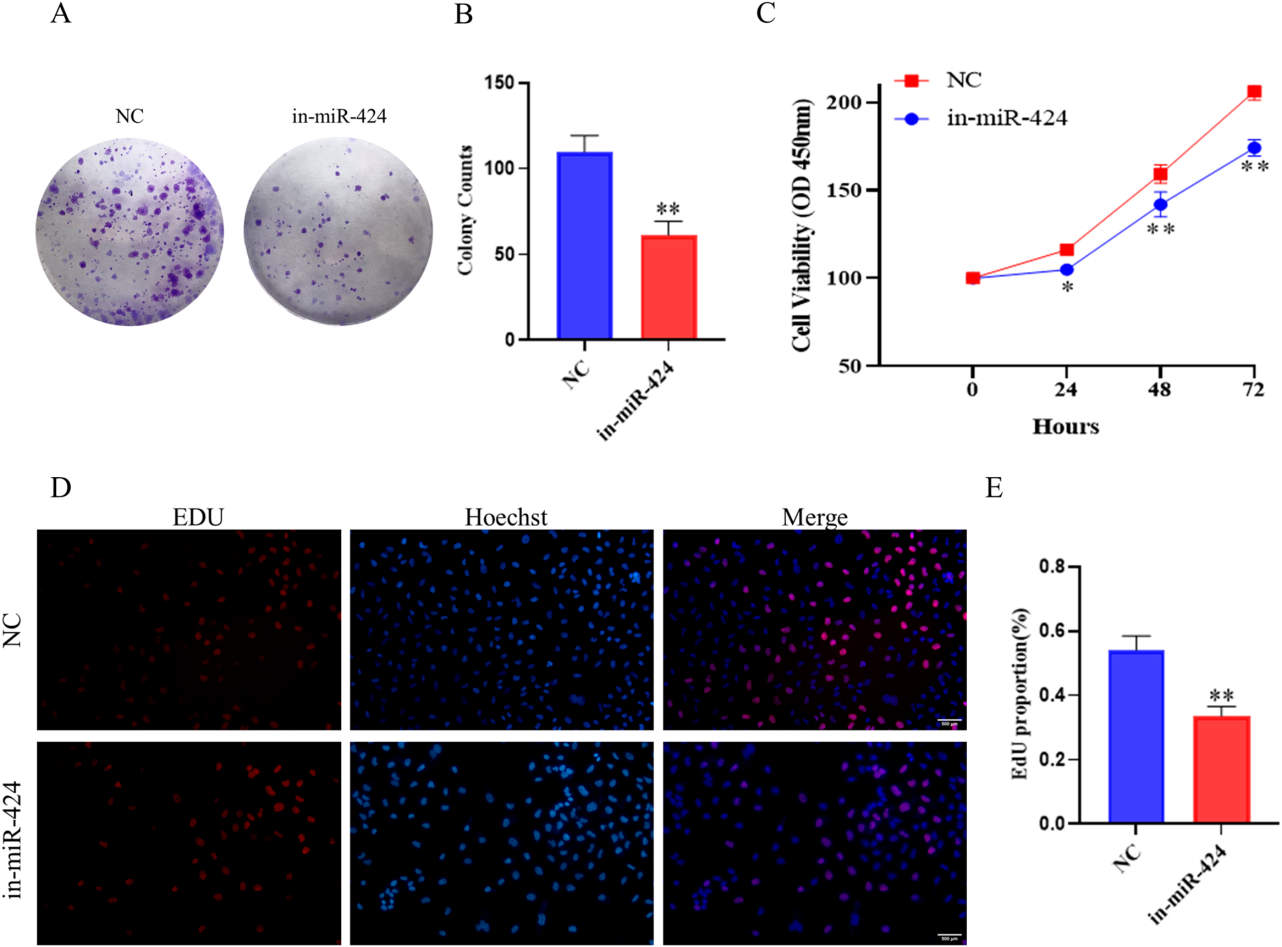
Effect of miR-424 inhibition on proliferation and colony formation. **(A)** Representative images of colony formation assay. **(B)** Quantification of colony numbers. Data are mean ± SD(n=3). ^**^p < 0.01. **(C)** Cell viability measured by CCK-8 assay. Data are mean ± SD(n=3). ^*^p < 0.05 and ^**^p < 0.01. **(D)** Representative images of EdU assay. Red: EdU-positive cells; blue: nuclei. **(E)** Quantification of EdU-positive cells. Data are mean ± SD(n=3). ^**^p < 0.01.

To further investigate the effect of miR-424-5p on Hep3B cells proliferation, we conducted experimental validation. The colony formation assay revealed that compared to NC group, the in-miR-424 group showed a significant reduction in the number of colonies formed (*P* < 0.01) (**Figures 4A, B**).

The CCK-8 assay further confirmed that cell viability was significantly decreased at 24, 48, and 72 h after miR-424-5p knockdown (*P* < 0.05 or *P* < 0.01) (**Figure 4C**).

Additionally, EdU staining results demonstrated a significant decrease in the proportion of EdU-positive cells in the in-miR-424 group compared to the NC group (*P* < 0.01) (**Figures 4D, E**).

These consistent results indicate that miR-424-5p plays a critical role in the proliferation of Hep3B cells, and suppressing miR-424-5p expression can effectively inhibit their proliferative capacity.

### 3.5 Bioinformatic prediction links miR-424-5p to cell cycle and PI3K/AKT signaling

**Figure 5:**
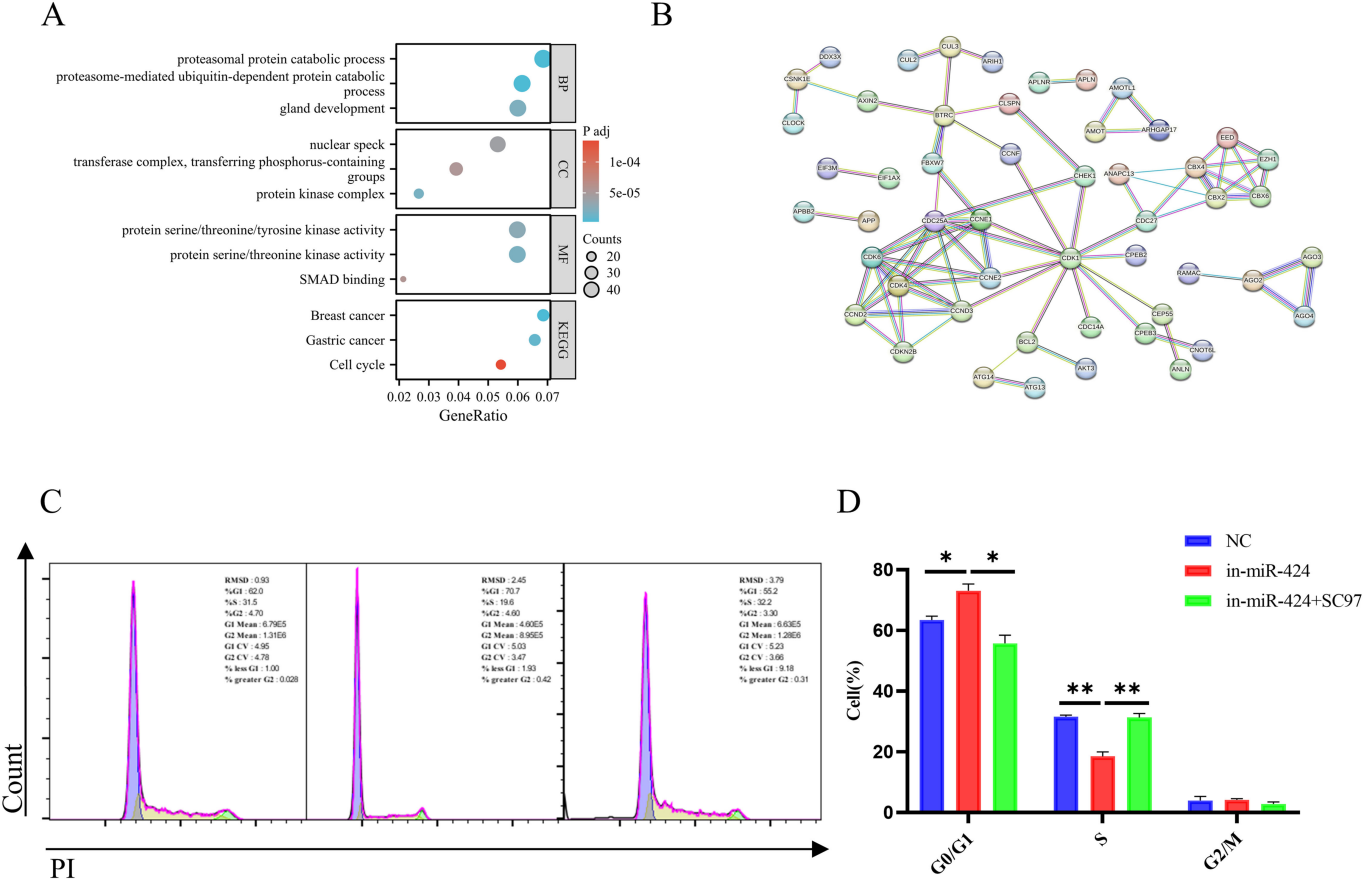
Mechanistic exploration of miR-424-5p affecting the Hep3B cell cycle through bioinformatic analysis. **(A)** Core nodes of the protein-protein interaction (PPI) network for miR-424-5p target genes. **(B)** GO and KEGG functional enrichment analysis of the predicted target genes. **(C, D)** Experimental results of miR-424-5p regulating the Hep3B cell cycle. Data are mean ± SD(n=3). ^*^p < 0.05 and ^**^p < 0.01.

To systematically elucidate the potential mechanism by which miR-424-5p promotes HCC progression, we performed bioinformatic analysis using the authoritative databases miRTarBase, TargetScan, and DIANA-microT-CDS, selecting genes common to all three. Construction of a protein-protein interaction network for these high-confidence targets revealed key nodes involved in cell cycle regulation, such as CCND1, CCNE1, and CDK1 (**Figure 5A**). GO and KEGG pathway enrichment analyses of the predicted targets showed significant enrichment in functional terms including “Cell cycle,” “Protein serine/threonine/tyrosine kinase activity,” and “Proteasomal protein catabolic process” (**Figure 5B**). Given that the PI3K/AKT pathway is a central regulator of cell cycle progression and protein kinase cascades, these bioinformatic findings strongly suggest that miR-424-5p may exert its oncogenic function by modulating key components within or upstream of the PI3K/AKT signaling axis (25–27).

To test this hypothesis, we further conducted cell cycle analysis. In Hep3B cells, knockdown of miR-424-5p significantly induced cell cycle arrest at the G0/G1 phase (P < 0.05), accompanied by a marked decrease in the proportion of S-phase cells (**Figures 5C, D**). Notably, when SC79, a specific AKT activator, was added to the miR-424-5p-knockdown cells, the G0/G1 phase arrest was partially reversed, and the S-phase cell proportion recovered to 35% (P < 0.01) (**Figures 5C, D**). This indicates that the regulatory effect of miR-424-5p on the cell cycle is likely dependent on PI3K/AKT pathway activity. These results provide direct rationale for our subsequent in-depth investigation into the PI3K/AKT pathway.

### 3.6 miR-424-5p regulates Hep3B cells functions via the PI3K/AKT signaling pathway

**Figure 6:**
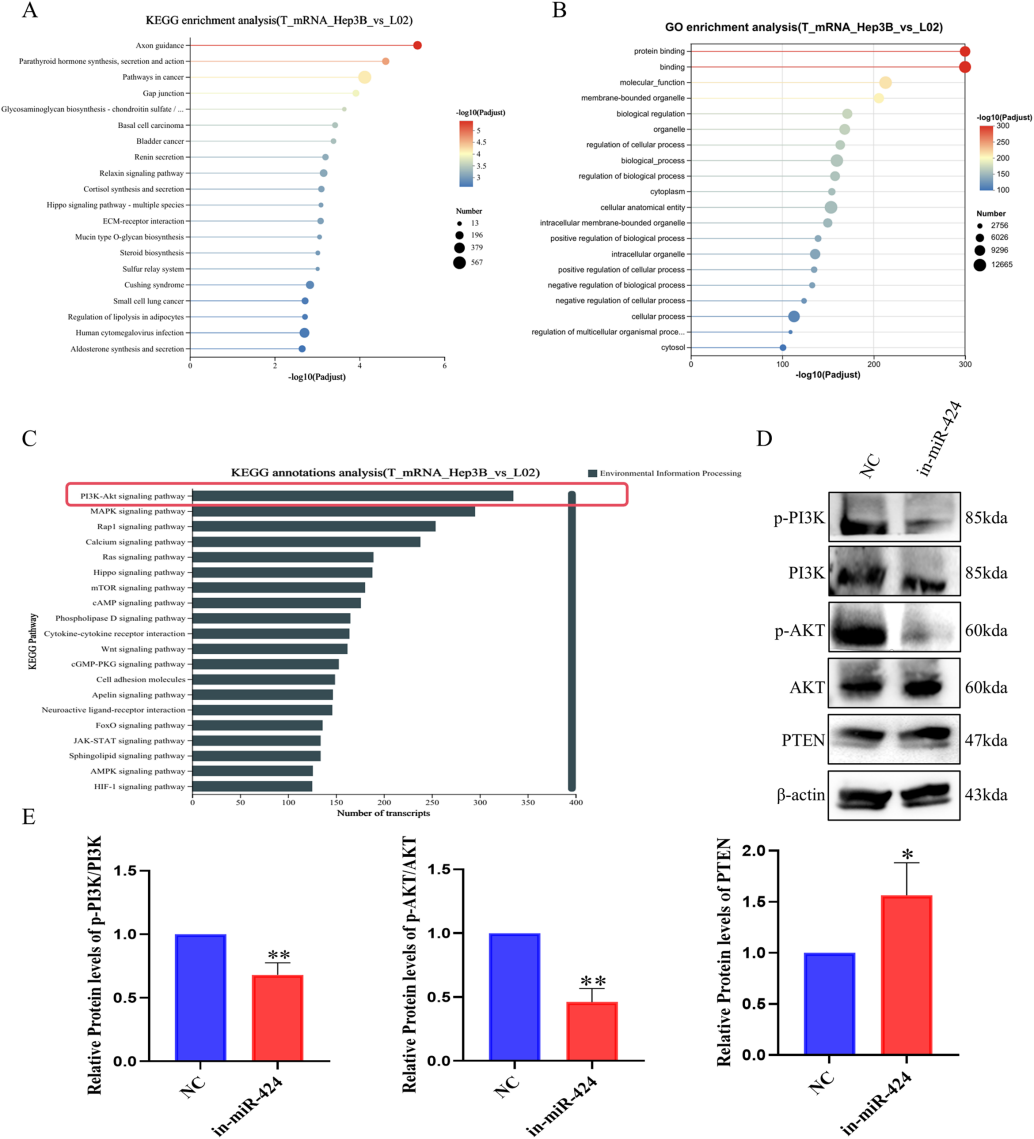
Effect of miR-424 on signaling pathways and related proteins in Hep3B cells. **(A)** KEGG pathway enrichment of DEGs between Hep3B and L02 cells. Dot size: number of genes; color: adjusted p-value. **(B)** GO enrichment of DEGs. Dot size: number of genes; color: adjusted p-value. **(C)** KEGG annotation showing transcript numbers per pathway. **(D)** Western blot of p-PI3K, PI3K, p-AKT, AKT, and PTEN in cells transfected with in-miR-424 and NC. *β*-actin was used as control. **(E)** Quantification of protein levels. Data are mean ± SD(n=3). ^*^p < 0.05 and ^**^p < 0.01.

To further investigate the mechanisms of miR-424-5p in HCC, we performed KEGG enrichment analysis following transcriptome sequencing. The results demonstrated significant enrichment of the target genes in pathways related to axon guidance, parathyroid hormone synthesis, gap junctions, and pathways in cancer (**Figure 6A**). GO analysis indicated that these genes were primarily involved in biological processes such as protein binding and molecular function (**Figure 6B**). Furthermore, KEGG annotation analysis revealed significant enrichment of these genes in the “PI3K-AKT signaling pathway” (**Figure 6C**, highlighted by a red box).

Subsequently, Western blot analysis confirmed that after miR-424-5p knockdown, the protein expression ratios of p-PI3K/PI3K and p-AKT/AKT were significantly decreased (*P* < 0.05), while the expression of the tumor suppressor gene PTEN was significantly upregulated (*P* < 0.01) (**Figures 6D, E**).

These results systematically reveal the molecular mechanism by which miR-424-5p affects the proliferation and migration of HCC cells through regulating the phosphorylation status of key molecules in the PI3K/AKT signaling pathway.

## 4 Discussion

This study elucidates a previously unknown oncogenic function of miR-424-5p in HBV-associated hepatocellular carcinoma. In contrast to its tumor-suppressive role in non-viral hepatocellular carcinoma contexts, our transcriptome analysis directly revealed significant enrichment of its target genes in the PI3K/AKT signaling pathway. This bioinformatic evidence was supported by functional experiments, which demonstrated that miR-424-5p drives cell cycle progression and proliferation in an AKT-dependent manner. Collectively, these data robustly substantiate that a functional switch of miR-424-5p occurs within the HBV infection context, whereby it assumes an oncogenic role by activating the PI3K/AKT signaling pathway.

HCC remains a major global health challenge with high morbidity and mortality (28). This study demonstrates that miR-424-5p is significantly upregulated in HBV-positive Hep3B cells (29, 30), a finding that contrasts with reports of its downregulation in non-specific or HBV-negative HCC contexts (31, 32). We further elucidated that this upregulation correlates with the activation of the PI3K/AKT signaling pathway, as evidenced by our functional assays and Western blot analysis. Our KEGG pathway analysis initially indicated this association, which was subsequently confirmed by the decrease in p-PI3K/PI3K and p-AKT/AKT ratios and the upregulation of PTEN upon miR-424-5p knockdown. These findings robustly demonstrate a previously unrecognized oncogenic role of miR-424-5p in this specific viral-associated context.

The most significant insight from our work is the etiology-dependent oncogenic function of miR-424-5p in HBV-HCC. The stark contrast between its role in our model and its tumor-suppressive function in other settings (33) can be attributed to the unique molecular landscape imposed by HBV infection. We propose that viral factors, particularly the HBx protein, act as a “molecular switch” that reprograms miR-424-5p’s function. This reprogramming may occur through multiple mechanisms (1): HBx may directly promote miR-424-5p transcription (34) (2); HBV-induced dysregulation of non-coding RNAs (e.g., circ-RNF13 (35)) could alter the competitive endogenous RNA (ceRNA) network, sequestering miR-424-5p and leading to its compensatory upregulation (3); The fundamental alteration of cellular signaling pathways by HBV may shift the functional outcomes of miR-424-5p target regulation. Our deliberate focus on the Hep3B model was therefore essential to uncover this virus-specific pathogenic axis, highlighting that miRNA function cannot be extrapolated without considering the underlying disease etiology.

While miR-424-5p is characterized as a tumor suppressor in many cancers (36), including non-HBV associated HCC (37), our findings robustly demonstrate an oncogenic role in the specific context of HBV-positive Hep3B cells. This etiology-dependent functional switch represents the most significant insight of our work.​ The consistent regulation of predicted target genes like STXBP5 and SYMPK, along with the functional validation of its impact on proliferation and migration, strongly supports that miR-424-5p function is reprogrammed within the HBV-altered molecular landscape.

Our data support a model where miR-424-5p activation contributes to HBV-HCC progression by attenuating PTEN and enhancing PI3K/AKT signaling, as evidenced by Western blot analysis (**Figures 6D, E**). Based on these findings and existing literature, we propose the “HBx-miR-424-5p-PI3K/AKT” axis as a plausible hypothesis for HBV-related hepatocarcinogenesis.​ However, it is crucial to state that our study does not provide direct experimental evidence that HBx protein transcriptionally upregulates miR-424-5p; this proposed upstream step requires future validation through chromatin immunoprecipitation (ChIP) assays or HBx-knockdown models. Nevertheless, the oncogenic function of the passenger strand miR-424-3p in other virus-associated cancers (38, 39) lends credence to the idea of a coordinated dysregulation of the miR-424/503 cluster in viral contexts.

This model underscores that miRNA function can act as a “molecular switch” dependent on specific etiology (36). The potential of targeting the miR-424-5p-PI3K/AKT axis, perhaps in combination with existing therapies, represents a promising avenue for future research in HBV-HCC treatment (40, 41).

Based on these findings, we propose the “HBx-miR-424-5p-PI3K/AKT” axis hypothesis, which offers a novel target for precision therapy in HBV-associated liver cancer. In clinical practice, combining miR-424-5p-targeting antisense oligonucleotides with PI3K inhibitors (e.g. LY294002) may yield enhanced therapeutic effects for HBV-positive patients.

However, this study has several limitations. First, the mechanistic pathway was analyzed in only one HCC cell line, and validation using additional HCC cell lines is necessary. Second, although our rescue experiment with SC79 supports the dependency of miR-424-5p function on PI3K/AKT pathway activation, whether PTEN is its direct target remains to be further validated by experiments such as dual-luciferase reporter assay. Third, whether HBx directly regulates the miR-424-5p promoter remains unclear. Future research should focus on constructing HBV transgenic mouse models to validate the virus dependent function of miR-424-5p *in vivo*, as well as screening for direct interactions between HBx and the miR-424-5p promoter using techniques such as ChIP assays (32).

In conclusion, this study elucidates the unique mechanism of miR-424-5p in HBV-HCC. Its upregulation and functional reversal may represent a critical step in HBx-mediated viral carcinogenesis. Further exploration of the clinical implications of etiology-specific miRNA regulatory networks is warranted.

## Data Availability

The original contributions presented in the study are publicly available. This data can be found here: https://www.ncbi.nlm.nih.gov/geo/; GSE316568.

## References

[1] Pellat A Barat M Coriat R Soyer P Dohan A . Artificial intelligence: A review of current applications in hepatocellular carcinoma imaging. Diagn Interv Imaging. (2023) 104:24–36. doi: 10.1016/j.diii.2022.10.001, PMID: 36272931

[2] Brown ZJ Tsilimigras DI Ruff SM Mohseni A Kamel IR Cloyd JM . Management of hepatocellular carcinoma: A review. JAMA Surg. (2023) 158:410–20. doi: 10.1001/jamasurg.2022.7989, PMID: 36790767

[3] Qiu H Zhang Y Xu F Xue S . Different nucleos(t)ide analogs in resected hepatitis B virus-associated hepatocellular carcinoma: a systematic review. Front Pharmacol. (2025) 16:1647888. doi: 10.3389/fphar.2025.1647888, PMID: 41322294 PMC12660110

[4] Dong Z Ye Q Zhou Y Shao Y Chen J Cai J . The LUBAC subunit HOIL-1 promotes the progression of HBV-associated hepatocellular carcinoma independently of linear ubiquitination. Exp Mol Med. (2025) 57:2317–30. doi: 10.1038/s12276-025-01556-4, PMID: 41053347 PMC12586429

[5] Ding F Zhong Y Zhang H Zhang D Zheng Z Zhang X . Abnormal levels of miRNA in pancreatic cancer are linked to tumor progression by regulating the translation of tumor-associated mRNA. Ann Med. (2025) 57:2541315. doi: 10.1080/07853890.2025.2541315, PMID: 40844410 PMC12377092

[6] Wang Y Fu Y . Identification of circRNA-miRNA-mRNA networks to explore underlying mechanism in lung cancer. Health Inf Sci Syst. (2025) 13:5. doi: 10.1007/s13755-024-00318-2, PMID: 39676897 PMC11645342

[7] Lai Y Peng Z He Z Lu Z Yan S Nie Q . Dioscin initiates dual roles in bladder cancer progression via miR-195-5p/FASN/SLC3A2 axis-mediated cell death mechanisms. Transl Oncol. (2025) 61:102534. doi: 10.1016/j.tranon.2025.102534, PMID: 40961580 PMC12476075

[8] García-Castillo J Martínez-Cáceres CM Bernabé-García M Munitiz V Ruiz De Angulo D Parrilla P . MicroRNA 196a contributes to the aggressiveness of esophageal adenocarcinoma through the MYC/TERT/NFκB axis. Mol Oncol. (2025) 19:3305–24. doi: 10.1002/1878-0261.70048, PMID: 40955778 PMC12591326

[9] Mnyandu NZ Limani SW Ely A Wadee R Arbuthnot P Maepa MB . Long-term inhibition of Hepatitis B virus gene expression by a primary microrna expressing ancestral adeno-associated viral vector. Virol J. (2025) 22:41. doi: 10.1186/s12985-025-02662-5, PMID: 39962472 PMC11834259

[10] Chen Y Yang X Feng M Yu Y Hu Y Jiang W . Exosomal miR-223-3p from bone marrow mesenchymal stem cells targets HDAC2 to downregulate STAT3 phosphorylation to alleviate HBx-induced ferroptosis in podocytes. Front Pharmacol. (2024) 15:1327149. doi: 10.3389/fphar.2024.1327149, PMID: 38444939 PMC10912342

[11] Liu J Kong L Bian W Lin X Wei F Chu J . circRNA_0001006 predicts prognosis and regulates cellular processes of triple-negative breast cancer via miR-424-5p. Cell Div. (2023) 18:7. doi: 10.1186/s13008-023-00089-4, PMID: 37194024 PMC10186655

[12] Wei B Xiao S Lou W . In silico whole-transcriptome analysis reveals a potential hsa_circ_0000375-miR-424-5p-TPM2/SRPX/SRGAP1 regulatory network related to liver metastasis of colorectal cancer. Heliyon. (2023) 9:e21688. doi: 10.1016/j.heliyon.2023.e21688, PMID: 37954397 PMC10638074

[13] Li Y Zhang C Zhao Z . CircSLCO3A1 depletion ameliorates lipopolysaccharide-induced inflammation and apoptosis of human pulmonary alveolar epithelial cells through the miR-424-5p/HMGB3 pathway. Mol Cell Toxicol. (2023) 19:107–18. doi: 10.1007/s13273-023-00341-6, PMID: 37359246 PMC10211294

[14] Wan MF Yang N Qu NY Pan YY Shan YQ Li P . MiR-424 suppressed viability and invasion by targeting to the DCLK1 in neuroblastoma. Eur Rev Med Pharmacol Sci. (2020) 24:5526–33. doi: 10.26355/eurrev_202005_21338, PMID: 32495887

[15] Lai JC Yang B Lee HW Lin H Tsochatzis EA Petta S . Non-invasive risk-based surveillance of hepatocellular carcinoma in patients with metabolic dysfunction-associated steatotic liver disease. Gut. (2025) 74:2050–7. doi: 10.1136/gutjnl-2025-334981, PMID: 40541300

[16] Zhang Y Li T Guo P Kang J Wei Q Jia X . MiR-424-5p reversed epithelial-mesenchymal transition of anchorage-independent HCC cells by directly targeting ICAT and suppressed HCC progression. Sci Rep. (2014) 4:6248. doi: 10.1038/srep06248, PMID: 25175916 PMC4150107

[17] Piao L Wang F Wang Y Yang Z Li Q Cui L . miR-424-5p regulates hepatoma cell proliferation and apoptosis. Cancer Biother Radiopharm. (2019) 34:196–202. doi: 10.1089/cbr.2018.2625, PMID: 30676784

[18] Chun HS Lee M Lee HA Park ES Choi JY Baek HS . A novel risk prediction model for hepatocellular carcinoma in MASLD: A multinational, multicenter cohort study. Clin Gastroenterol Hepatol. (2025). doi: 10.1016/j.cgh.2025.06.025, PMID: 40645391

[19] Sugiyama G Nio K Okada H Kida A Sako K Iwata Y . Vascular endothelial growth factor receptor 2-targeted therapy suppresses the progression of alpha-fetoprotein-positive hepatocellular carcinoma after combination therapy with anti-programmed death-ligand 1 and anti-vascular endothelial growth factor-A antibodies. Gastro Hep Adv. (2026) 5:100778. doi: 10.1016/j.gastha.2025.100778, PMID: 41140754 PMC12546878

[20] Kumada T Toyoda H Yasuda S Koshiyama Y Ito T Akita T . Hepatitis B virus RNA predicts hepatocellular carcinoma despite viral suppression. Aliment Pharmacol Ther. (2025). doi: 10.1111/apt.70483, PMID: 41328676 PMC13021286

[21] Wang F Li J Huang Y Yan F Gao H Zhou W . CAR-T cell engineered with TCR-like antibody specific for HBV surface antigen epitope E183-91/HLA-A *0201 exhibit potent activity against HBV-HCC. Oncoimmunology. (2025) 14:2546404. doi: 10.1080/2162402x.2025.2546404, PMID: 40820888 PMC12363532

[22] Wang XQ Fan YQ Hou DX Pan CC Zheng N Si YQ . Establishment and validation of diagnostic model of microvascular invasion in solitary hepatocellular carcinoma. J Invest Surg. (2025) 38:2484539. doi: 10.1080/08941939.2025.2484539, PMID: 40254744

[23] Malekan M Dozandeh-Jouybari A Joudaki N Ahangari M Valadan R Asgarian-Omran H . Inhibition of PI3K/AKT/mTOR signaling enhances autophagy in HL-60 acute myeloid leukemia cells: An integrative bioinformatic and *in vitro* study. Biochem Biophys Rep. (2025) 44:102220. doi: 10.1016/j.bbrep.2025.102220, PMID: 40917720 PMC12409794

[24] Zhai M Xue H Li F . PAR2 regulates proliferation, migration of lung cancer and chemotherapy sensitivity by involving PTEN pathway. Future Sci OA. (2025) 11:2535221. doi: 10.1080/20565623.2025.2535221, PMID: 40686241 PMC12296048

[25] Zhang R Yin X Xu Z Yan Q . Novel Ce/Cu-modified black ceramics disrupted mitochondrial dysfunction and tricarboxylic acid cycle to induce cuproptosis in osteosarcoma cells. Biomater Adv. (2026) 180:214576. doi: 10.1016/j.bioadv.2025.214576, PMID: 41218466

[26] Jing J Chen S Wu X Yang J Liu X Wang J . Recombinant tissue plasminogen activator protects neurons after intracerebral hemorrhage through activating the PI3K/AKT/mTOR pathway. Neural Regener Res. (2026) 21:1574–85. doi: 10.4103/nrr.Nrr-d-23-01953, PMID: 39104167 PMC12407559

[27] Guo Y Wang F Wang B Zhou Y Wang C Hu T . ACE-mediated glycosylation stabilizes PSAP to promote GPR37-dependent macrophage-nucleus pulposus cells crosstalk and TGFβ Signaling in alleviating intervertebral disc degeneration. Adv Sci (Weinh). (2025) 12:e10662. doi: 10.1002/advs.202510662, PMID: 41085015 PMC12631909

[28] Filho AM Laversanne M Ferlay J Colombet M Piñeros M Znaor A . The GLOBOCAN 2022 cancer estimates: Data sources, methods, and a snapshot of the cancer burden worldwide. Int J Cancer. (2025) 156:1336–46. doi: 10.1002/ijc.35278, PMID: 39688499

[29] Anwanwan D Singh SK Singh S Saikam V Singh R . Challenges in liver cancer and possible treatment approaches. Biochim Biophys Acta Rev Cancer. (2020) 1873:188314. doi: 10.1016/j.bbcan.2019.188314, PMID: 31682895 PMC6981221

[30] Ding Z Wang L Sun J Zheng L Tang Y Tang H . Hepatocellular carcinoma: pathogenesis, molecular mechanisms, and treatment advances. Front Oncol. (2025) 15:1526206. doi: 10.3389/fonc.2025.1526206, PMID: 40265012 PMC12011620

[31] Elhefnawi M Salah Z Soliman B . The promise of miRNA replacement therapy for hepatocellular carcinoma. Curr Gene Ther. (2019) 19:290–304. doi: 10.2174/1566523219666191023101433, PMID: 31657677

[32] Majumdar S Chakraborty A Das S Gorain M Chatterjee S Dey I . Sponging of five tumour suppressor miRNAs by lncRNA-KCNQ1OT1 activates BMPR1A/BMPR1B-ACVR2A/ACVR2B signalling and promotes chemoresistance in hepatocellular carcinoma. Cell Death Discov. (2024) 10:274. doi: 10.1038/s41420-024-02016-0, PMID: 38851743 PMC11162467

[33] Chen RC Wang J Kuang XY Peng F Fu YM Huang Y . Integrated analysis of microRNA and mRNA expression profiles in HBx-expressing hepatic cells. World J Gastroenterol. (2017) 23:1787–95. doi: 10.3748/wjg.v23.i10.1787, PMID: 28348484 PMC5352919

[34] Kim S Park J Han J Jang KL . Hepatitis B Virus X Protein Induces Reactive Oxygen Species Generation via Activation of p53 in Human Hepatoma Cells. Biomolecules. (2024) 14:1201. doi: 10.3390/biom14101201, PMID: 39456134 PMC11505488

[35] Chen Y Li S Wei Y Xu Z Wu X . Circ-RNF13, as an oncogene, regulates Malignant progression of HBV-associated hepatocellular carcinoma cells and HBV infection through ceRNA pathway of circ-RNF13/miR-424-5p/TGIF2. Bosn J Basic Med Sci. (2021) 21:555–68. doi: 10.17305/bjbms.2020.5266, PMID: 33714261 PMC8381212

[36] Ghafouri-Fard S Askari A Hussen BM Taheri M Akbari Dilmaghani N . Role of miR-424 in the carcinogenesis. Clin Transl Oncol. (2024) 26:16–38. doi: 10.1007/s12094-023-03209-2, PMID: 37178445 PMC10761534

[37] Topisirovic I Sonenberg N . mRNA translation and energy metabolism in cancer: the role of the MAPK and mTORC1 pathways. Cold Spring Harb Symp Quant Biol. (2011) 76:355–67. doi: 10.1101/sqb.2011.76.010785, PMID: 22123850

[38] Xia Y Han B Zhang F Li Q Feng Q Zhang S . Pae/exo at PF-127 promote diabetic wound healing through miR-424-5p. Phytomedicine. (2025) 142:156688. doi: 10.1016/j.phymed.2025.156688, PMID: 40347888

[39] Shan J Pu J Chen X Zhang Y Li J Qin L . CircRNA circACTN4 Promotes the Progression of Epithelial-Mesenchymal Transition in Hepatocellular Carcinoma by Targeting the miR-424-5p/NCAPG/Wnt Axis. Clin Exp Med. (2025) 25:47. doi: 10.1007/s10238-025-01573-7, PMID: 39891781 PMC11787268

[40] Chen W Lin C Wang L Yu Z Xu Y Zhang M . Hepatitis B virus X protein promotes the progression and immune escape of hepatocellular carcinoma by activating KLF16-C12orf49-PD-L1 axis. Oncogene. (2025) 44:4747–62. doi: 10.1038/s41388-025-03625-4, PMID: 41238928

[41] Feng YF Su TM Hu BB Wang H Li QM Yin QB . Diagnostic performance of serum origin recognition complex subunit 1 protein for hepatitis B virus-related hepatocellular carcinoma. World J Gastroenterol. (2025) 31:112481. doi: 10.3748/wjg.v31.i44.112481, PMID: 41356527 PMC12678938

